# Increasing the Reliability of Simulation Tests in Navigation and Maneuvering Simulators Using the k-Epsilon Model Based on the RANS Method

**DOI:** 10.3390/s21154995

**Published:** 2021-07-23

**Authors:** Krzysztof Czaplewski, Slawomir Swierczynski, Piotr Zwolan

**Affiliations:** 1 Department of Navigation, Faculty of Navigation, Gdynia Maritime University, 81-225 Gdynia, Poland; 2 Department of Navigation and Hydrography, Polish Naval Academy, 81-127 Gdynia, Poland; s.swierczynski@amw.gdynia.pl (S.S.); p.zwolan@amw.gdynia.pl (P.Z.)

**Keywords:** maritime navigation, computer simulation, computational fluid dynamics

## Abstract

The influence of wind on the maneuverability of sea-going vessels is a known factor limiting their maneuverability, especially in the case of very large vessels. Adverse weather conditions often limit the maneuverability of vessels or even make it impossible to enter the port. This results in longer delivery times for transported goods as well as measurable material losses for both carriers and their owners. This situation is often caused by a lack of information on differences in the prevailing weather conditions at the entrance to the port and at the seaport itself. There are simulation tools, such as the methods of computational fluid dynamics (CFD), which, after their appropriate adaptation and use in a virtual environment, have become important decision-making tools supporting the port administration when deciding about the movement of vessels. In this article, the authors present the results of research aimed at adapting one of the CFD methods for the needs of maritime navigation. The effects of the work were verified in a virtual environment and were successfully implemented in the port waters of Gdansk, Poland.

## 1. Introduction

The issue of the influence of wind on the behavior of a vessel in sea areas is documented in the subject-related literature. The most frequently examined research problem is its impact on the marine environment and maritime transport as a function of its direction and speed. The results of the analyses can be found in [1,2], for example. Hydro-meteorological conditions, which are interdependent, significantly influence the development of maritime transport. This problem is described even in extremely rarely frequented parts of the globe such as the Arctic; the problems in these latitudes are described in [3]. Wind also determines the level of safety in air transport; the problems in air navigation are outlined, for example, in [4]. Collecting long-term measurement data and analyzing their impact on various forms of navigation is very costly, and in the case of preparing port infrastructure construction projects, it is often not sufficient for their proper development. Therefore, modern navigation and maneuvering simulators have become the basic tool supporting design works. The design of a new port infrastructure is always verified in a virtual environment, as iterated in [5,6,7]. The authors also used this tool during the navigational analysis of the construction of a footbridge in Gdansk (Poland), as can be found in [8], or during the preparation of the virtual model of the Deep Water Container Terminal in Gdańsk. The results of using the navigation simulator are presented in [9]. However, the area of the authors’ interest includes not only the environmental conditions for the functioning of sea transport, but also the construction of virtual models of real vessels in the navigation simulator environment. In order to create a virtual model of a vessel, it is also necessary to understand its susceptibility to meteorological conditions, including the effects of wind [10].

The issue of the influence of wind on a vessel’s behavior is an important aspect during the implementation of simulation tests. Proper vessel drift assessment for simulated weather conditions has a key impact on safe navigation. This is a particularly important factor when maneuvering in a port and on approach to ports. Currently, a common approach is the use of navigation and maneuvering simulators to assess the correctness of design assumptions for newly designed and reconstructed port waters, as outlined in [11]. Such projects require the implementation of detailed navigational analyses, one of the elements of which are simulation studies. Their goal may be, among other aspects, to determine the maximum dimensions of a vessel that can safely maneuver in a selected body of water [12]. Modern navigation and maneuvering simulators used during analyses made in port waters most often do not take into account the detailed impact of port buildings and other onshore infrastructure on the disturbance of the wind field. They use simple air flow models for coastal areas and buildings. Detailed turbulence models are included only for the calculation of forces between vessels [13]. To ensure a high level of credibility of simulation tests, maneuvering data should be recorded on real vessels, the virtual equivalents of which are used during the tests. For this purpose, one can use measurement sets that provide necessary data for analyses. For example, the set described in [14] allows the data necessary for the adaptation of the virtual vessel model that is available in the simulator environment for the purposes of design analyses to be obtained. At the stage of conducting analyses in the simulator environment, one should also have detailed knowledge about the distribution of the wind field. This is especially important during tests inside the port basin, where buildings and port infrastructure have a significant impact on the weakening of the wind field, and thus the change of the vessel’s maneuvering parameters. Currently, research teams most often use the approach of considering only the basic wind model available in simulators during research. The simulation assumes a constant wind speed and direction [15], which is in line with the current requirements. However, this approach does not provide real knowledge about the possibility of maneuvering vessels in the area under research, because the vessel behaves differently in open water and in the port under the same hydro-meteorological conditions. During this research, the aim of which was to determine the impact of buildings on the change of wind speed and direction in the Kashubian Canal in Gdansk (Poland), the authors decided to apply an innovative approach to the implementation of simulation studies, with a particular focus on navigational analyses. The effects of the work are the content of this article, which presents the use of CFD applications [16]. The use of this application allows us to obtain a detailed distribution of the wind field in the research area under consideration. The stages comprising the creation of three-dimensional models of port facilities and vessels [8,10] are very important elements of this process. In order to verify the possibility of using the CFD module, a number of real studies were carried out with the use of our own measurement platform [17], the results of which were compared with simulation studies. The results of the conducted tests and final analyses made it possible to develop new port procedures and increase the parameters of vessels that can operate at Gdansk Bulk Terminal (GBT) in the Kashubian Canal in Gdansk.

## 2. General Characteristics of Computational Fluid Dynamics

Computational fluid dynamics is a computer method of analysis that uses numerical methods and enables the fast, efficient, and accurate simulation of fluid flow and heat transfer in complex geometric systems [18]. Due to the discretization and numerical solution of partial differential equations describing the flow, it is possible to approximate the distribution of velocity, pressure, temperature, and other parameters in the flow. CFD methods are successfully used in numerical analyses with the aim of better understanding complex phenomena related to fluid movement. Thus, CFD methods are also widely used in engineering design in order to improve existing solutions or to create new ones [18]. Computational fluid dynamics is a branch of fluid mechanics that uses numerical analysis and algorithms to solve problems related to fluid flows. High-performance computers are used to make calculations required to simulate the interaction of liquids and gases with surfaces defined by boundary conditions. CFD is based on the Navier–Stokes equations. The equations resulting from the application of Newton’s second law to fluid motion, together with the assumption that the stress in a fluid is the sum of the diffusive viscous component and the pressure component, describe how the velocity, pressure, temperature, and density of a moving fluid are correlated [16].

Through CFD analysis, we can understand the flow and heat transfer throughout the design process. The basic methodology of any engineering CFD analysis is based on several procedures [16]:

- Understanding the flow model;
- Checking the adopted model;
- Model optimization.

Numerical fluid flow simulations are now widely used in various fields of science. Research and engineering teams use CFD in the following applications, among others [19,20,21,22,23,24]:

- Forecasting weather and warning against natural disasters;
- Vehicle design—improving aerodynamic properties;
- Designing the environment in an energy-saving and safe manner;
- Design and maintenance of pipeline networks in an optimal manner;
- Treatment and prevention of diseases, including diseases of the veins.

Commercially available CFD calculation software uses several main flow modeling methods. For the present research problem, the most reliable models are as follows:

- Large-eddy simulation (LES) and detached-eddy simulation (DES)
  The LES method allows us to adjust the precision of calculations to the problem undergoing research. Using this method, we obtain a numerical mapping of the wind effect on structures closest to the real impact. This method simulates vortices of a size similar to the size of the mesh element of the model grid. The basic limitations of the LES method are the computational capabilities of computers and time consumption.
- Reynolds-averaged Navier–Stokes solvers (RANS)
  The most frequently used method in calculations is the RANS method (in particular, the k-ε turbulence model), which is much faster [25]. It is a mathematical model based on average variable values for both steady state and dynamic flows. The RANS model can be used when simple buildings are considered.

There are many computer programs available to solve the research problems related to CFD. These programs, depending on their complexity, can be free or commercial. However, when generalizing the way these applications work, the process of solving the problem is very similar.

**Figure 1:**
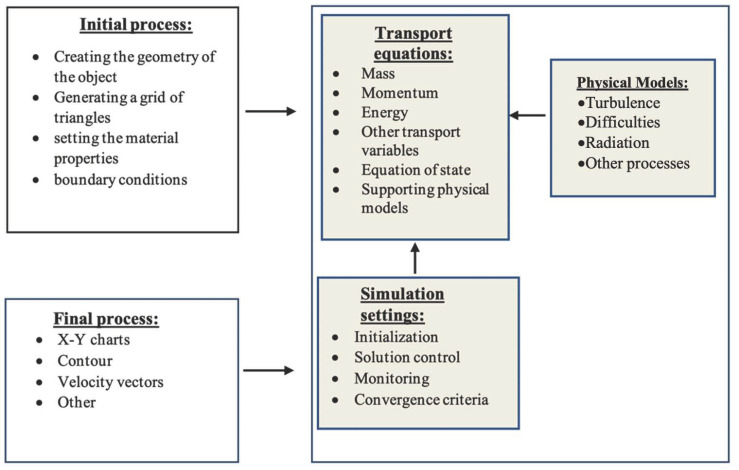
The simulation diagram in ANSYS Fluent.

Figure 1 shows a simulation scheme using the ANSYS Fluent computer application [26] consisting of three basic elements of computer calculations.

Many turbulence models are used when using CFD applications to calculate fluid flow or air mass distribution. Basically, these models are classified according to the main equation and the numerical method. The most commonly used models of turbulence in computer applications are as follows:

- k-epsilon (k-ε);
- k-omega (k-ω);
- SST;
- SSG Reynolds.

As presented, for example in [27], the k-epsilon (k-ε) turbulence model is the most commonly used model in computational fluid dynamics (CFD) and is used to simulate average flow characteristics for fully turbulent flow conditions. Therefore, this model was used in this research project. The model is based on two equations; in the first of which the transported variable is turbulent kinetic energy (k). In the second equation, however, the transport variable is the dissipation rate of turbulent kinetic energy (ε). It is worth mentioning that the model uses wall functions and is characterized by good convergence. It has low efficiency for complex flows with strong pressure gradients or a complex flow path. The model is highly developed and accurate despite many limitations. It includes sub-models to take into account compressibility, elasticity, etc. They are a great advantage of this model. It is worth mentioning that it is suitable for complex flows involving rapid changes in fluid parameters, moderate turbulence, or local disturbances (e.g., the detachment of the boundary layer, and turbulence behind flowing bodies).

The k-omega (k-ω) model is one of the most commonly used models of turbulence. It is a two-equation model, meaning that it contains two additional transport equations to represent turbulent flow properties. As in the k-epsilon turbulence model, wall functions are used here, and there is good convergence. Another advantage of this model is its high accuracy for internal flows and curves. The k-ω turbulence model is characterized by high efficiency compared with the models from the k-ε group, in terms of simulating the phenomena that occur in the boundary layer, flow disturbances, and flows at low Reynolds numbers. The k-omega model includes sub-models that take into account the compressibility of fluids and the transition state between laminar and turbulent flow, often used in the aerospace industry and rotating machinery (publication).

Menter’s shear stress transport model (SST) is a combination of two models: k-ε (in the outer boundary layer region) and k-ω (in the inner boundary layer region). It consists of two equations and is commonly referred to as a hybrid model due to the combination of two models. The shear stress transport formula (SST) combines the best features of the two components of this combination. It is a smooth transition from the standard k-ω model used in the boundary layer to the k-ε model as it moves away from the flow-limiting surface. The use of the k-ω formula in the inner parts of the boundary layer makes the model fit directly to the wall through a viscous sublayer; therefore, the k-ω SST model can be used without any additional damping functions [20]. The k-ω SST model unfortunately produces excessively high levels of turbulence in regions with high normal stress, such as stagnant regions and regions with high acceleration. This tendency is admittedly much less distinct than in the case of the normal k-ε model. It contains a modified turbulent viscosity formulation to account for the transport effect in terms of major shear stresses.

## 3. The Concept of Using CFD in Navigation and Maneuvering Simulators

Currently, navigation and maneuvering simulators are not only devices used to train crews of vessels; one of the other ways in which they are used is research related to the broadly understood human activity in marine areas. Most often, the main area of research using simulators is work related to maneuvering a vessel in specific weather conditions. Therefore, it can be concluded that one of the most important elements influencing the credibility of the results obtained during the research is the correctly defined wind field distribution in the studied area. This is especially important during research into the behavior of a vessel in harbor basins. When maneuvering the vessel in the port, one must comply with the applicable port regulations. This mainly concerns the permissible hydro-meteorological conditions for the entry of vessels into the port (e.g., wind force and waves). These regulations do not always reflect the actual conditions inside the port basin, as the wind speed is measured on the harbor master’s tower. Measurement sensors located on the harbor master’s tower are placed at high altitudes. Due to this fact, the measured wind speed does not take into account the influence of buildings. Thanks to the achievements of computational fluid dynamics (CFD), it is possible to estimate the wind speed fields between buildings for selected heights above sea level in the port basin. This knowledge greatly increases the awareness of the actual conditions prevailing in the various locations of the port basin in relation to the wind measurement on the harbor master’s tower. This aspect affects the safety of navigation in the port and is an important factor enabling permission to be granted to enter the port. Currently, there are often situations where permission is not granted, despite the fact that the conditions inside the port allow for safe maneuvering, which is a huge loss for port wharf operators. Knowledge about the exact distribution of the wind field may form the basis for the implementation of navigation analyses with the use of navigation and maneuvering simulators. Currently, a common approach is to simulate constant conditions inside port basins (available in the simulator software), while it is reasonable to carry out simulation tests in conditions as close to real as possible. An additional way to use CFD in a virtual environment may be to simulate the distribution of sea currents in selected waters. Currently, simplified models are used that define only the direction and speed of the current on the basis of information, often archival, regarding the available data. CFD calculation applications significantly help in accurately determining the distribution of sea currents, especially in areas covered by the modernization of port infrastructure.

Research teams use navigation and maneuvering simulators to carry out navigation analyses. The navigational analysis is a detailed analysis of the issues of maneuvering a vessel during its approach and entry, as well as the departure from the offshore structure and entry and exit from the port basin and the port. Its purpose is often to decide on the choice of a project and to build new port quays or modernize them. When adapting CFD methods to simulation tests, the methodology of their implementation should be modified. In such a case, the following steps should be taken when carrying out such tests:

- Real field tests, measuring the direction and speed of the wind and, if necessary, the parameters of the sea current, as a reference value;
- Building a three-dimensional model of the port area for its implementation into CFD software;
- The implementation of the model into the CFD program and execution of computer simulations for the measured real conditions;
- The analysis of the performed simulations in terms of the consistency of real and simulated tests;
- The implementation of the results obtained from the CFD application into the navigation and maneuvering simulator software in the form of precise weather zones.

The results obtained from the research using the above methodology make it possible to verify the assumptions made during the CFD analysis by comparing them with the actual research. If the obtained results are consistent, one can easily modify the weather parameters in the CFD software in order to obtain results for other weather parameters. An advantage of this approach is that there is no need for additional real research. This methodology increases the level of credibility of research in navigation and maneuvering simulators compared with the use of basic simplified weather models used in simulators. In the further part of the article, the authors present the application of the proposed methodology when developing a navigational analysis for the needs of the operator of a grain terminal in the port of Gdansk.

## 4. Construction of a Measuring Set for Real Measurements

**Figure 2:**
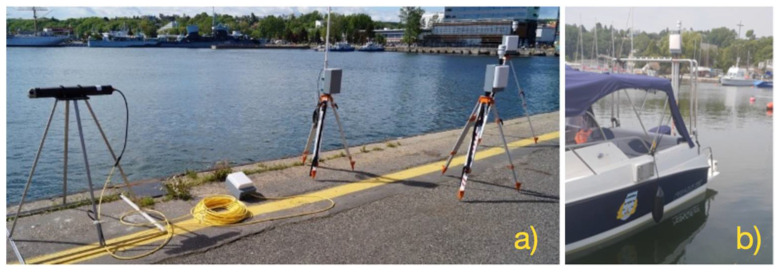
Weather data recording system: (**a**)—during static test, (**b**)—before dynamic test.

In order to obtain real measurement data, it is necessary to have an appropriate measurement set. A device must enable the registration of actual hydrometeorological conditions in selected parts of the water area at the same time. An important element of this process is the timing of the measurements for the purpose of their later comparison with the results obtained outside the port. For the purposes of this research, the authors of the article designed and built a set of mobile meteorological stations. The principle of the system is based on radio data transmission from the measuring stations to the receiving station. Figure 2 shows elements of the system used during statistic and dynamic tests.

**Figure 3:**
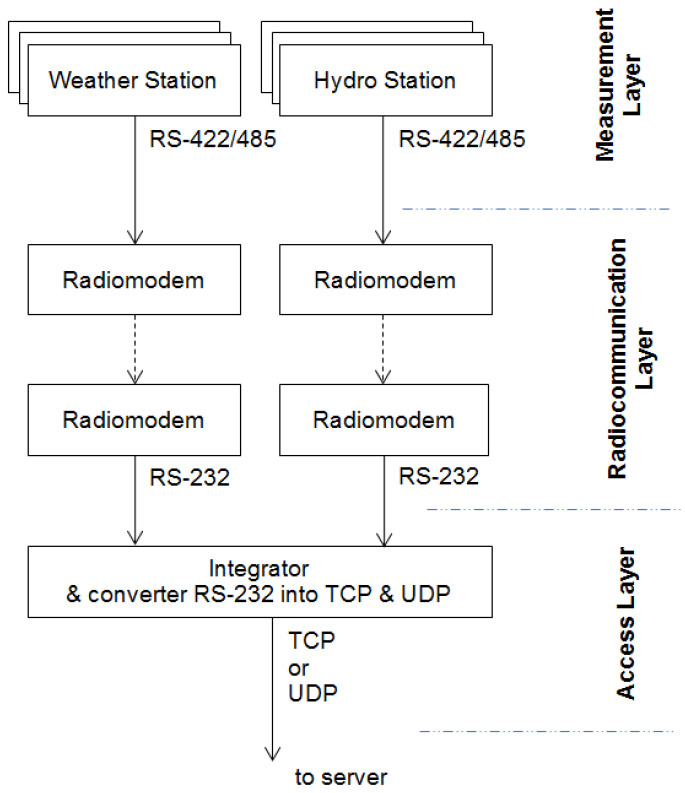
Block diagram of the measurement system.

The developed weather data recording system enables both static and dynamic measurements. The main sensor for measuring the weather is the AIRMAR 150WX sensor. This model is recommended for moving applications in which the real and apparent winds are different. It includes additional sensors such as a 10 Hz GPS receiver, which gives the course over ground (COG), speed over ground (SOG) and position a solid-state two-axis compass and a three-axis accelerometer for pitch and roll, as well as a field-replaceable relative humidity sensor. It is equipped with configurable digital data outputs RS232, RS422, and the controller area network (CAN BUS), ensuring universal weather monitoring. Figure 3 shows a diagram of the weather data recording system.

**Figure 4:**
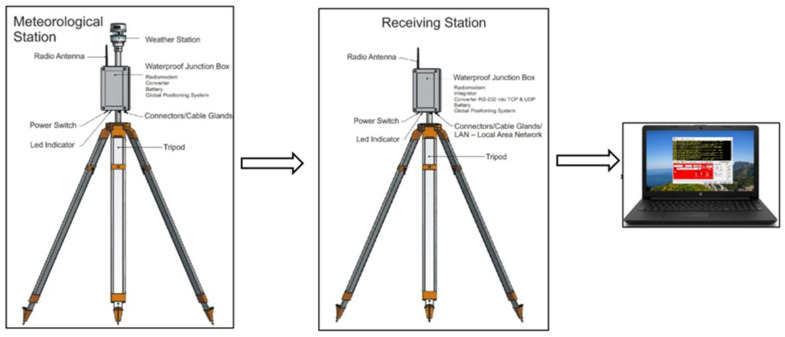
Method of data transmission.

The data recorded by the weather station are transmitted to the receiving station. Measurement data are recorded with the use of the “RealTerm” tool, which is a specialized application for capturing, controlling, and debugging complex data streams, both binary and of other types. The method of data transmission is shown in Figure 4.

## 5. Testing the Vessel’s Behavior in a Navigation and Maneuvering Simulator with the Use of CFD

In order to use the CFD application for wind field modeling in the environment of the navigation and maneuvering simulator, the research process should consist of the following:

- The registration of actual weather data in a selected water area;
- The preparation of a three-dimensional model of the researched water area for the needs of its implementation into the CFD software;
- The performance of a simulation of wind field distribution in the CFD application for input data reflecting actual registrations;
- The implementation of the obtained CFD results in the environment of the navigation and maneuvering simulator in the form of weather zones;
- The implementation of simulation tests in a navigation and maneuvering simulator.

**Figure 5:**
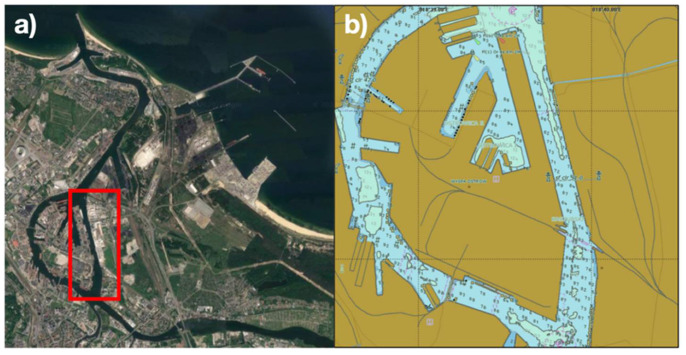
Area accepted for research: The Kashubian Canal ((**a**)—https://www.google.pl/maps, access date: 20 May 2021; (**b**)—NT Pro 5000 simulator software).

The research area in this article is the Kashubian Canal, located in the port of Gdansk (Figure 5). The city of Gdansk is located in the Gulf of Gdansk, Poland. The characteristic features of this climate are low annual temperature amplitudes, delayed seasons, an extension of the period between summer and winter, strong winds—mainly from the west—and the occurrence of sea breezes. The change of the spatial arrangement, resulting in this case from the development of the area in the immediate vicinity of the North Peninsular Breakwater, requires the determination of a number of parameters regarding the interaction of wind with engineering structures. The starting point for the analysis of the wind field at the entrance to the port basin is the situation at the entrance to the port. In the studied area, the largest percentage share, as well as the greatest force, is generated by winds from S to N directions from the west [28].

**Figure 6:**
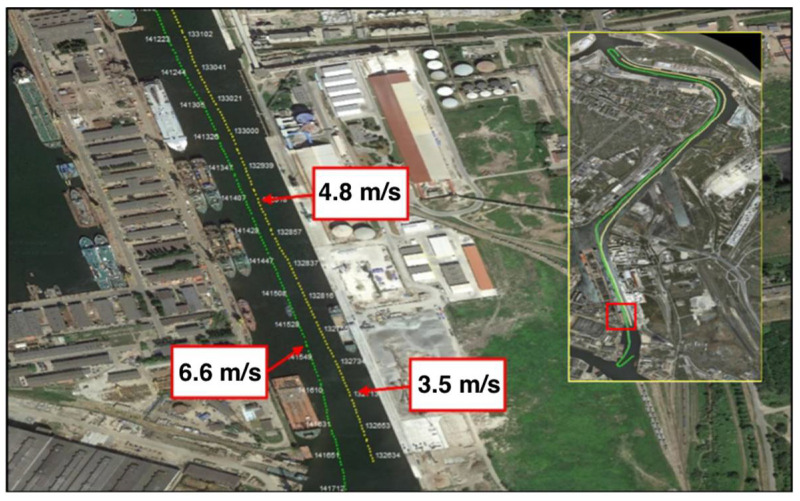
View of the passage during dynamic tests with individual measurement positions.

In order to obtain real weather data, a number of both dynamic and static measurement sessions were performed. Dynamic tests were conducted in which the direction and speed of the actual wind were recorded while maneuvering the vessel on the route Harbor Master’s Office, Bytomskie Quay, Gdansk. Then, wind speeds and directions were compared in individual port locations with the data recorded on the Harbor Master’s Office tower which defines the conditions, allowing the ship to enter the port. Data were recorded on the vessel at second intervals using the meteorological station, as described in point 4. Figure 6 shows a map with the vessel’s route marked as measurement points. Each of these points visualizes the position of the vessel to which the measured wind speed and direction was assigned.

Table 1 shows the results of the selected measurement session recorded during the maneuvering of the vessel. The data in Table 1 present information about the measured wind direction and speed in comparison with the data received from the service at the Harbor Master’s Office.

**Figure 7:**
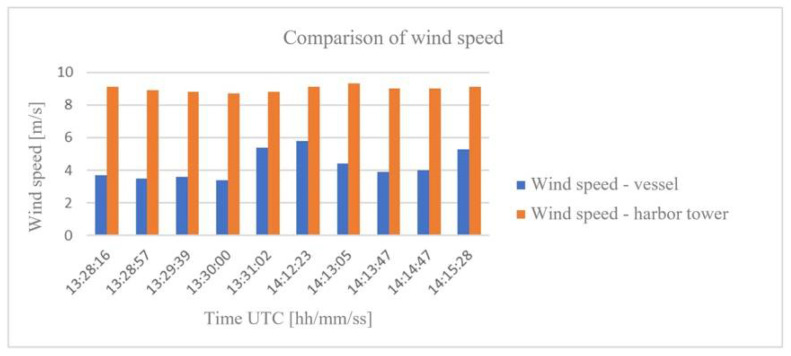
Comparison of wind speed from the vessel with the Harbor Master’s Office tower.

Figure 7 shows a comparison of the results of actual measurements made at the Harbor Master’s Office tower with the actual measurements made on the vessel during the tests.

Additionally, long-term stationary measurement sessions were carried out at the port wharf in order to obtain more detailed information about the weather conditions prevailing during the mooring of vessels. Table 2 presents the general results of the comparative analysis of the wind speed recorded at the entrance to the port and at the quay for selected hours during a three-day measurement session.

After the real research was completed, the next step was to prepare a three-dimensional model of the Kashubian Canal area for the purposes of its implementation into the CFD software. This process can be performed in several ways:

- With the use of ready-made models available in the service databases for the needs of, for example, spatial planning;
- On the basis of plans, maps, and satellite photos in computer-aided design (CAD) applications;
- Based on data obtained from devices—i.e., a laser scanner or drone;
- Creating a new, non-existent model from scratch in a CAD-type application.

**Figure 8:**
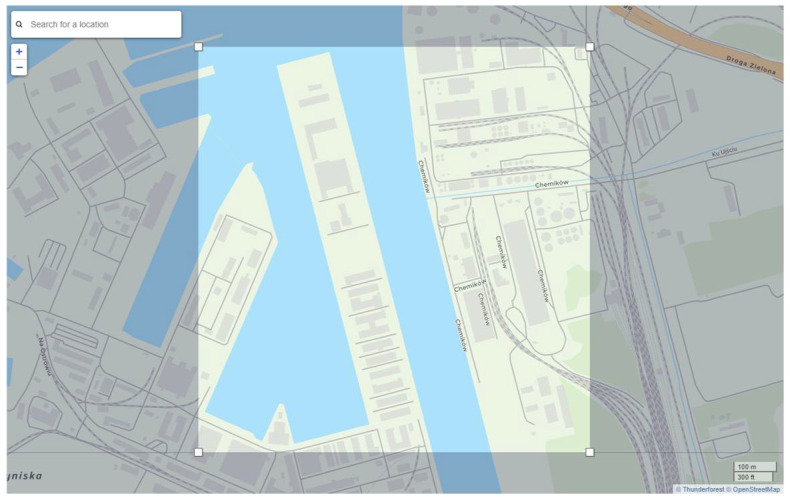
Area of interest presented in the Cadmapper application.

The research described in this article used the Cadmapper application, which transforms data from public sources such as OpenStreetMap, the National Aeronautics and Space Administration (NASA), and the United States Geological Survey (USGS) into structured AutoCAD files. The first stage of the work was to define the area of interest in the Cadmapper application, which included the Kashubian Canal (Figure 8).

**Figure 9:**
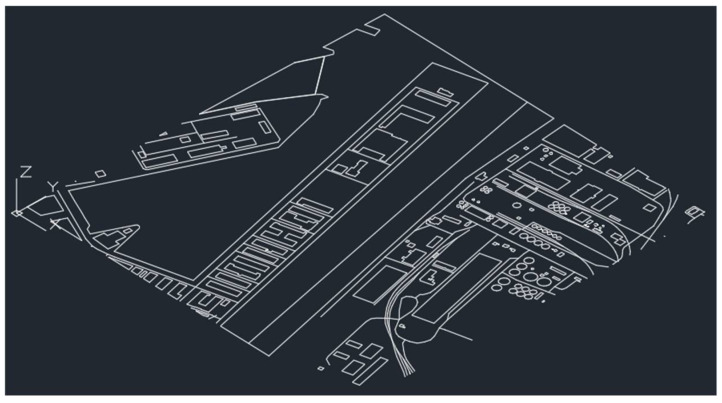
Sketch of the area created in the AutoCAD software.

Then, the set area was exported to the *.dxf extension supported by the AutoCAD platform. The AutoCAD software processed the sketch of the area by removing unnecessary layers, such as the road layer, railway layer, and power network layer, the geometry of which did not affect the results of the analyses (Figure 9).

**Figure 10:**
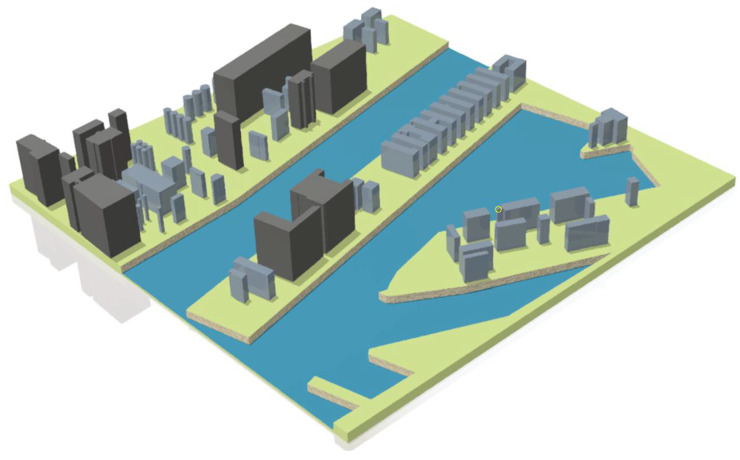
Virtual model of the Kashubian Canal.

In the next step, a three-dimensional model was built. In the CAD environment, on the previously created sketch, three-dimensional elements were created by simple and compound construction extrudes. Appropriate heights of the buildings were given and 2 m high quays were introduced (Figure 10).

The last stage of creating the three-dimensional model was exporting the file to the Standard for the Exchange of Product Model Data (STEP), which complies with the ISO 10303 standard. The model prepared in this way was ready to be implemented in the CFD simulation program.

**Figure 11:**
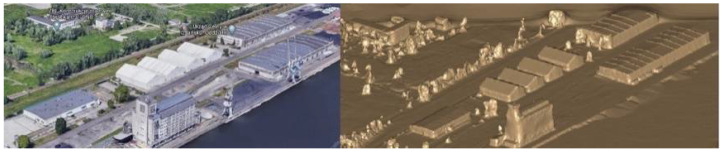
Numerical model of terrain coverage converted to the 3D model in Cyclone 3DR software.

In the event that it is necessary to have very detailed models of objects, the Numerical Model of Terrain Coverage from state resources can be used. The downloaded data in ASCII XYZ GRID formats can be converted, e.g., in the Cyclone 3DR application to obtain object models for analysis (Figure 11).

After the three-dimensional model of the water area was prepared, it was introduced into the CFD software. During the research work, the ANSYS CFD software was used. However, all simulation software based on the finite element method for computational fluid dynamics (CFD) designs uses a similar formula for solving computational problems. The differences arise only from the selection of the appropriate type of solving model used in the flow analysis.

**Figure 12:**
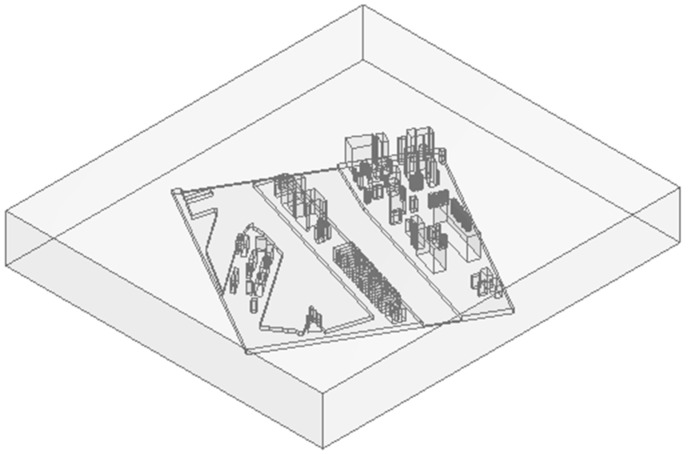
The function of rotating the CAD model.

The simulation started with the creation of the design diagram using the “Fluid Flow” tool with the “Fluent” calculation model. In the first stage, a previously prepared three-dimensional CAD model was selected and introduced. Using the “Design Modeler” tool, an aerodynamic calculation tunnel was created and planes were defined that were responsible for the direction of the wind flow. Then, using the rotation function, the CAD model was set in the appropriate position to the angle of the wind direction (Figure 12).

**Figure 13:**
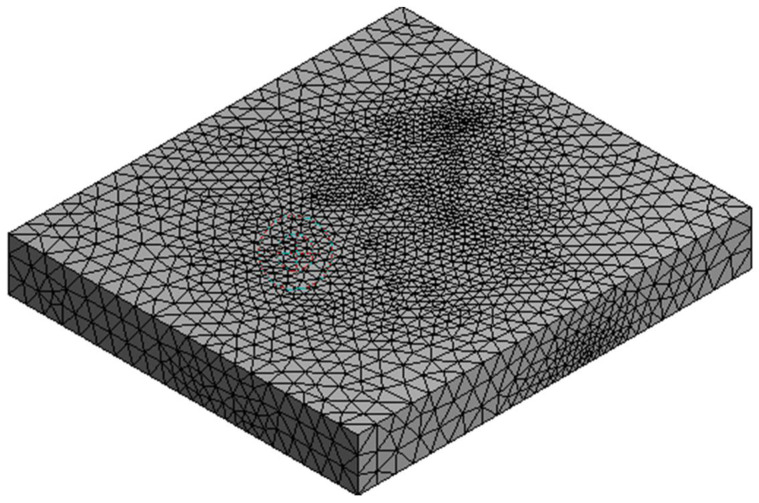
The effect of model discretization—computational grid.

In the next step, the entire model was discretized using the “Auto Mesh” tool, thanks to which a mesh based on automatic sizing was obtained (Figure 13). This type of mesh is more intelligent because the size of the elements vary and fit the surface of the model better.

The configuration of the simulation began with the selection of the k-epsilon turbulence model described earlier. The parameters introduced in the simulation software and general constants adopted in the computing environment were used. Then, in the simulation project settings, air properties were given: a density of 1.225 kg/m^3^ and a dynamic viscosity of 0.17894 kg/m·s. The next step was to introduce the boundary conditions for the previously created planes, where the initial wind speed of 9 m/s was assigned to the air inlet plane. This was the average wind speed recorded at the Harbor Master’s tower. Project initialization started by entering criteria into the software computing cache. In the final stage, a computer simulation was started, yielding results that were useful for the entire project.

**Figure 14:**
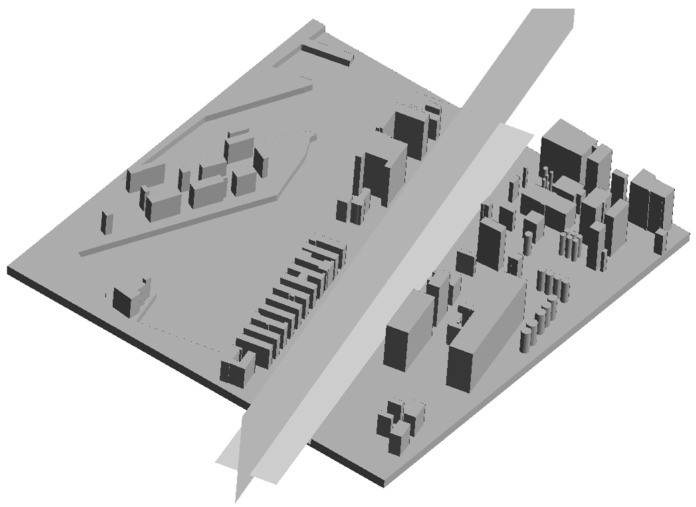
Auxiliary planes visualizing the wind field distribution.

In order to visualize the distribution of the wind field in the horizontal and vertical planes accurately, the auxiliary planes shown in Figure 14 were created.

**Figure 15:**
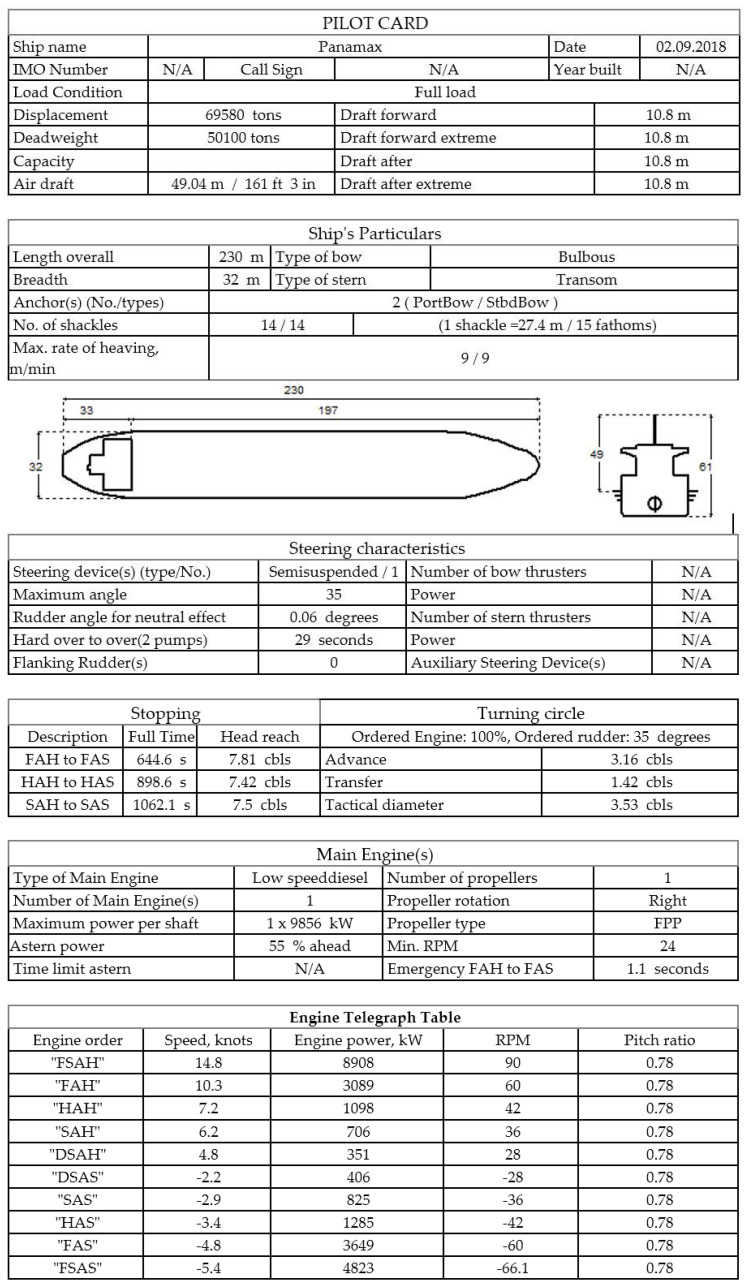
Pilot card of the vessel used during the research.

The final stage of the simulation tests was the implementation of the results obtained from the CFD application into the navigation and maneuvering simulator environment. The presented methodology was used in the research work, the purpose of which was to determine safe wind conditions enabling the approach of the PANAMAX class vessel to the GBT (Gdansk Bulk Terminal) in the port of Gdansk. The maneuvering data of the unit are shown in Figure 15.

The simulation tests included towing a ship in the Kashubian Canal. Several simulation tests were carried out for various weather conditions. The tests were conducted for the classic weather zone available in the simulator, and the zone improved with the results obtained from the CFD application.

## 6. Results of Simulation Analyses

**Figure 16:**
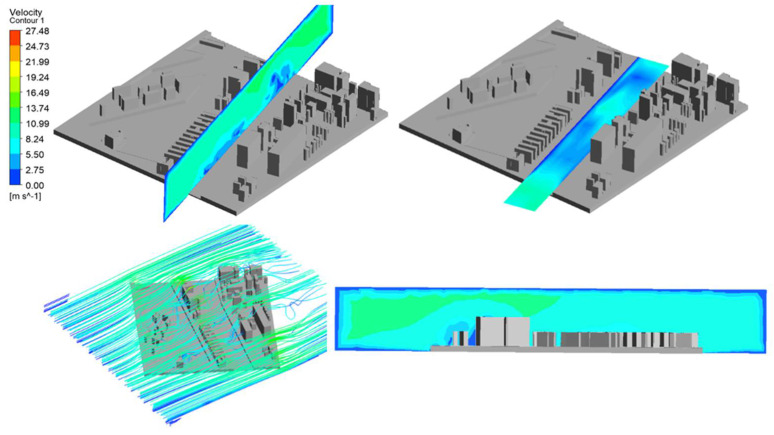
Simulation results presented by means of a contour map.

After making all the configurations and setting the input parameters, such as the boundary conditions in the simulation program, the simulations were performed. The input parameters of the wind in terms of its direction and speed were the values entered from the “port” column, which reflected the wind measurements at the Harbor Master’s Office tower, without taking into account the port buildings. The simulation results are presented in Table 3 and illustrated in Figure 16.

Figure 16 shows the simulation results for the vertical and horizontal planes. They were prepared using a contour map.

**Figure 17:**
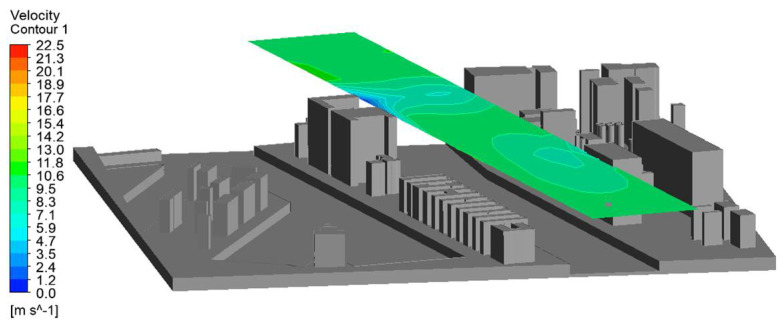
The simulation results presented for the horizontal plane at the height of 15 m, whereas Table 4 shows the values for the simulation at a height of 15 m.

In order to obtain the most reliable results regarding the use of CFD methods in supporting navigational analyses, additional computer simulations were performed. The simulations were performed to illustrate the wind speed at a different height, and the value adopted during the configuration was the height of 15 m because this is the height of the buildings in the simulated harbor area (Figure 17). The intention was that the wind speed at the height of 15 m, despite the unchanging nature of the buildings of the port, should differ from that calculated on the ground. In this way, another aspect of the problem was highlighted. Vessels moving in the port area have different windage areas depending on their height, which in turn translates into their variable susceptibility to wind in the same wind field. The methodology of using the CFD application enables us to obtain information about the vertical distribution of air masses, which is presented in the figure below, showing the wind speed in relation to the previous simulations.

**Figure 18:**
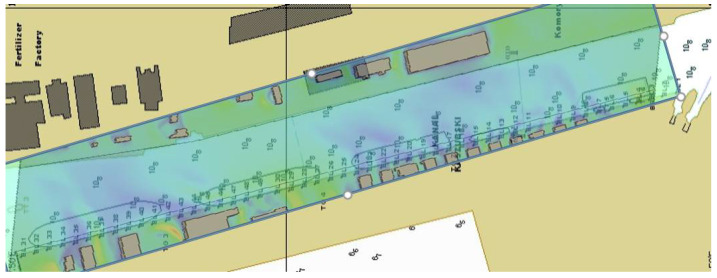
The process of creating a weather zone.

The final stage of the work was the implementation of the results obtained from the CFD simulation into the simulator environment. This was achieved by creating a weather zone characterized by a detailed distribution of the wind field. Figure 18 shows a fragment of the simulation scenario with the texture applied to create the weather zone.

**Figure 19:**
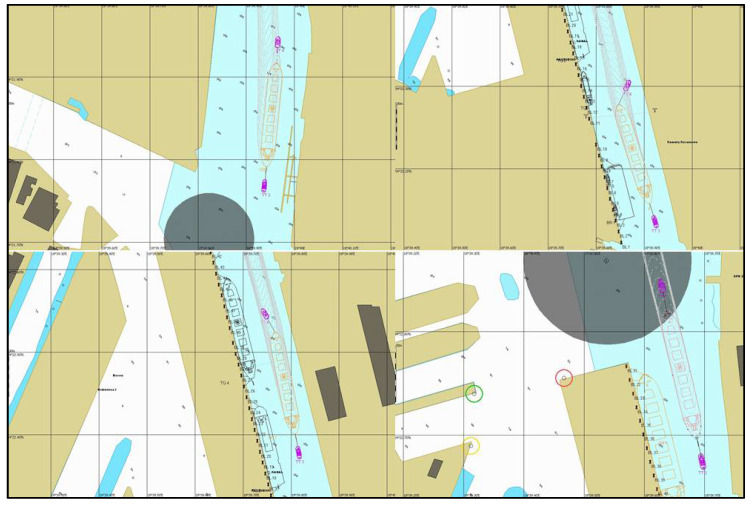
Simulation of a vessel towing in the Kashubian Canal.

**Figure 20:**
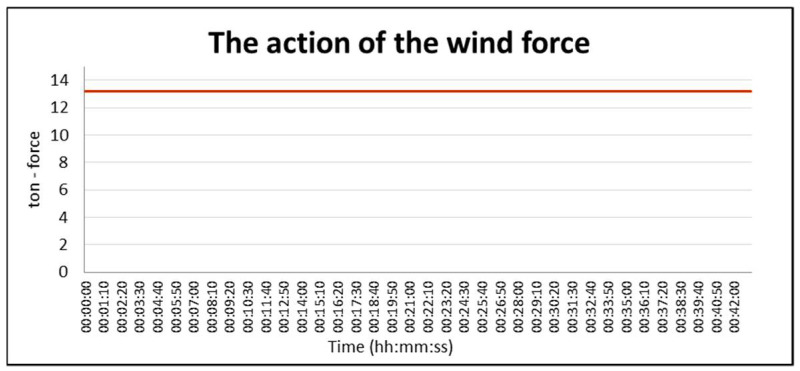
The force of wind pressure on the vessel’s hull for constant parameters of the weather zone (1 ton force = 9.80665 kN in SI system).

**Figure 21:**
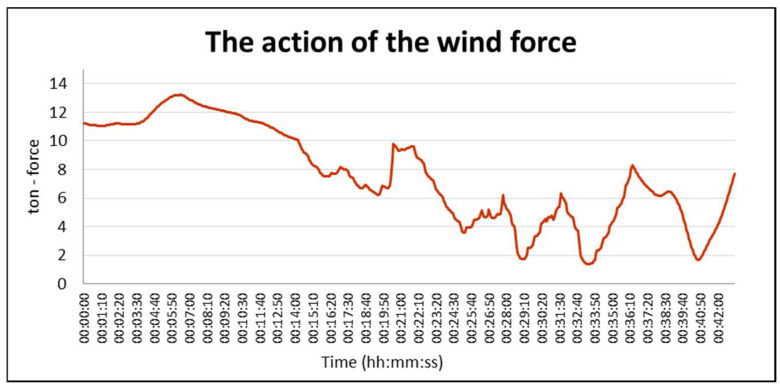
The force of wind pressure on the vessel’s hull for a detailed distribution of the wind field (1 ton force = 9.80665 kN in SI system).

The prepared weather zone was used during simulation tests of the entry of a PANAMAX vessel to the port of Gdansk. The results of one of the series of simulations made for the purposes of the navigational analysis carried out for the Kashubian Canal at the Port of Gdansk are presented below [29,30]. The graphics show the course of the simulation (Figure 19) and the distribution of forces acting on the hull of the vessel towed through the Kashubian Canal (Figure 20 and Figure 21).

Figure 20 shows the distribution of forces acting on the hull without a specific weather zone; i.e., for a constant wind direction and speed. Due to the lack of variability of the wind parameters, the wind pressure force was constant at about 13 tons for a vessel, moving at a constant towing speed and a constant course.

Figure 21 shows the results of simulation tests for the prepared weather zone with a detailed distribution of the wind field. The curve shows the influence of the weather zone on the weakening of the wind field, i.e., the impact of buildings on the change of wind parameters. The graphic analysis shows the significant drops in wind pressure to values below 2 tons. This has a significant impact on the maneuverability of the vessel and the work of the tugs.

As can be seen in the figures above, there are significant differences in the force of the wind pressure on the vessel according to different parameters of the weather zone. The use of detailed weather information increases the realism of simulation research, especially when conclusions from simulation studies form the basis for building new port infrastructure. The use of CFD applications in modeling weather conditions in navigation and maneuvering simulators may be increasingly widely used in order to increase the level of navigation safety and the reliability of simulation tests.

## 7. Conclusions

The conducted research confirmed the discrepancy between wind field distribution in sea areas directly adjacent to sea ports and their counterparts in port waters. This situation often causes the port administration to refuse to maneuver large vessels in ports despite favorable weather conditions. Port regulations concerning port traffic usually relate to measurements taken at port entry heads and often restrict their operation. While such a situation is appropriate in small ports, it unnecessarily limits their work in ports with extensive infrastructure and in many port basins.

The use of a simple-to-use computer simulation can improve the port’s operation while ensuring an appropriate level of navigation safety in port basins.

The adaptation of the computational fluid dynamics methods proposed in this article gives the opportunity to present real weather conditions in port waters and can be a useful tool to support the decision-making process and improve the work of large seaports.

The theses made during the research were verified in a navigation and maneuvering simulator by implementing a detailed wind field distribution. The results of the simulation tests showed a satisfactory level of consistency of the wind speed results calculated by the application with the real measurements. This proves the possibility of using the CFD module for the implementation of simulation tests; in particular, navigation analyses.

The results of the simulation studies were implemented in the port of Gdansk, which led to a change in port regulations [29,30]. The change in the regulations made it possible to handle larger vessels by the port, which translated into an increase in the tonnage handled and a reduction in the time of reloading goods.

The use of CFD applications for modeling weather conditions in navigation and maneuvering simulators should be extended in order to increase the level of navigation safety and increase the attractiveness of seaports as important maritime transport hubs. As a result, this will not only accelerate the flow of increasing amounts of goods by sea, but it will also have a positive impact on the natural environment by reducing the pollution generated by other forms of transport.

The next stages of research currently being carried out by the authors are focused on a wider (than described in this article) use of CFD for navigational analyses as a function of the hydrological conditions prevailing in coastal sea and port areas.

## Figures and Tables

**Table 1:** Actual wind measurements.

Time (UTC + 1)	Vessel		Port	
	Wind Direction [°]	Wind Speed [m/s]	Wind Direction [°]	Wind Speed [m/s]
13:28:16	255.0	3.7	243.0	9.1
13:28:57	256.0	3.5	244.0	8.9
13:29:39	258.0	3.6	244.0	8.8
13:30:00	256.0	3.4	243.0	8.7
13:31:02	251.0	5.4	245.0	8.8
14:12:23	250.0	5.8	245.0	9.1
14:13:05	260.0	4.4	247.0	9.3
14:13:47	257.0	3.9	249.0	9.0
14:14:47	255.0	4.0	249.0	9.0
14:15:28	255.0	5.3	248.0	9.1

**Table 2:** Recorded wind directions and speeds.

Time (UTC +1)	V Port	V GBT	Direction	Direction	Speed Difference
	m/s	m/s	Port	GBT	m/s
08:00:00	4.0	2.1	S	226.0	1.9
12:00:00	6.5	3.2	S	231.0	3.3
16:00:00	7.0	4.5	SSW	233.3	2.5
20:00:00	12.0	9.0	SSW	243.0	3.0
00:00:00	7.0	5.3	SSW	199.3	1.7
04:00:00	10.0	3.3	SSW	195.3	6.7
08:00:00	9.3	6.0	SW	253.5	3.3
12:00:00	12.0	6.6	SW	245.8	5.4
16:00:00	9.0	2.0	SW	212.4	7.0
20:00:00	5.4	3.0	S	191.0	2.4
00:00:00	5.3	1.2	S	219.8	4.1
04:00:00	5.3	1.3	S	098.2	4.0
08:00:00	5.0	1.7	SE	213.3	3.3
12:00:00	7.0	4.6	S	240.1	2.4

**Table 3:** CFD simulation results for the height of the port wharf.

Time (UTC + 1)	Vessel		Port		Results of CFD Simulation	
	Wind Direction [°]	Wind Speed [m/s]	Wind Direction [°]	Wind Speed [m/s]	Wind Direction [°]	Wind Speed [m/s]
13:28:16	255.0	3.7	243.0	9.1	240.0	3.5
13:28:57	256.0	3.5	244.0	8.9	246.0	3.3
13:29:39	258.0	3.6	244.0	8.8	249.0	3.5
13:30:00	256.0	3.4	243.0	8.7	246.0	3.2
13:31:02	251.0	5.4	245.0	8.8	240.0	5.1
14:12:23	250.0	5.8	245.0	9.1	240.0	5.6
14:13:05	260.0	4.4	247.0	9.3	249.0	4.5
14:13:47	257.0	3.9	249.0	9.0	249.0	4.0
14:14:47	255.0	4.0	249.0	9.0	240.0	4.1
14:15:28	255.0	5.3	248.0	9.1	240.0	5.5

**Table 4:** Comparison of CFD simulation.

Time (UTC + 1)	CFD Simulations for the Height of the Port Wharf		CFD Simulations for the Height of 15 m	
	Wind Direction [°]	Wind Speed [m/s]	Wind Direction [°]	Wind Speed [m/s]
13:28:16	240.0	3.7	251.0	9.8
13:28:57	246.0	3.5	251.0	7.3
13:29:39	249.0	3.6	251.0	7.8
13:30:00	246.0	3.4	255.0	8.7
13:31:02	240.0	5.4	250.0	9.8
14:12:23	240.0	5.8	250.0	10.2
14:13:05	249.0	4.5	255.0	9.4
14:13:47	249.0	3.9	250.0	8.8
14:14:47	240.0	4.1	250.0	9.2
14:15:28	240.0	5.5	250.0	9.9

## Data Availability

The data presented in this study is available upon request of the corresponding author. The data is not publicly available due to third party copyright. The measurements were conducted on behalf of a third party.
